# Effect of pulsed light on postharvest disease control-related metabolomic variation in melon (*Cucumis melo*) artificially inoculated with *Fusarium pallidoroseum*

**DOI:** 10.1371/journal.pone.0220097

**Published:** 2020-04-20

**Authors:** Francisco Oiram Filho, Ebenézer de Oliveira Silva, Mônica Maria de Almeida Lopes, Paulo Riceli Vasconselos Ribeiro, Andréia Hansen Oster, Jhonyson Arruda Carvalho Guedes, Dávila de Souza Zampieri, Patrícia do Nascimento Bordallo, Guilherme Julião Zocolo

**Affiliations:** 1 Department of Chemical Engineering, Science Center, Federal University of Ceará, Fortaleza, Ceará, Brazil; 2 Multiuser Laboratory of Natural Products Chemistry, EMBRAPA Agroindústria Tropical, Fortaleza, Ceará, Brazil; 3 Department of Biochemistry and Molecular Biology, Science Center, Federal University of Ceará, Fortaleza, Ceará, Brazil; 4 Post Harvest Laboratory, EMBRAPA Uva e Vinho, Bento Gonçalves, Rio Grande do Sul, Brazil; 5 Department of Analytical and Physical-Chemical Chemistry, Science Center, Federal University of Ceará, Fortaleza, Ceará, Brazil; 6 Department of Organic and Inorganic Chemistry, Science Center, Federal University of Ceará, Fortaleza, Ceará, Brazil; Universidade de Coimbra, PORTUGAL

## Abstract

Pulsed light, as a postharvest technology, is an alternative to traditional fungicides, and can be used on a wide variety of fruit and vegetables for sanitization or pathogen control. In addition to these applications, other effects also are detected in vegetal cells, including changes in metabolism and secondary metabolite production, which directly affect disease control response mechanisms. This study aimed to evaluate pulsed ultraviolet light in controlling postharvest rot, caused by *Fusarium pallidoroseum* in ‘Spanish’ melon, *in natura*, and its implications in disease control as a function of metabolomic variation to fungicidal or fungistatic effects. The dose of pulsed light (PL) that inhibited *F*. *pallidoroseum* growth in melons (*Cucumis melo* var. Spanish) was 9 KJ m^–2^. Ultra-performance liquid chromatography (UPLC) coupled to a quadrupole-time-of-flight (QTOF) mass analyzer identified 12 compounds based on tandem mass spectrometry (MS/MS) fragmentation patterns. Chemometric analysis by Principal Components Analysis (PCA) and Orthogonal Partial Least Squared Discriminant Analysis (OPLS-DA) and corresponding S-Plot were used to evaluate the changes in fruit metabolism. PL technology provided protection against postharvest disease in melons, directly inhibiting the growth of *F*. *pallidoroseum* through the upregulation of specific fruit biomarkers such as pipecolic acid (**11**), saponarin (**7**), and orientin (**3**), which acted as major markers for the defense system against pathogens. PL can thus be proposed as a postharvest technology to prevent chemical fungicides and may be applied to reduce the decay of melon quality during its export and storage.

## Introduction

Melon (*Cucumis melo* L.) is a widely produced fruit in different parts of the world and is an economically crucial part of Brazilian exports. However, a major fragility in the postharvest chain of melon is the incidence of postharvest pathologies, particularly rot caused by *Fusarium pallidoroseum*, which are responsible for postharvest melon losses. Melon is a ground plant whose fruit is in contact with the soil, thus facilitating its contamination with *F*. *pallidoroseum* [1]. *This fungal pathogen* is a widespread and common species in tropical, subtropical, and Mediterranean climates, often isolated from plants with complex diseases; it is also known to be toxigenic [2]. The losses caused by fungal diseases negatively affect the commercial balance around the world, where the phytosanitary barriers of some countries only allow the import of fruits with high biological control; therefore, these losses are important factors to be avoided in melon cultivation.

Fungal diseases from the genus *Fusarium* in fruit are traditionally controlled by the application of synthetic fungicides; this treatment leaves chemical residues, which could be harmful to the consumer and also encourages the development of fungicide-resistant strains of fungal pathogens. To overcome these challenges, several alternative or integrative approaches, including physical methods, are imperative to develop fruit with increased natural defense responses through the resistance mechanisms induced by abiotic stress [3].

Among these strategies, the application of technologies using light as an abiotic factor, which aims to promote a regulatory and signaling role in the developmental and metabolic processes of plants, is encouraged [4]. The application of ultraviolet light as a continuous low-intensity radiation (UV-continuous) is widely reported in the induction of resistance mechanisms and control of postharvest diseases, thus extending the shelf-life of fruit and vegetables [5, 6]. Some studies show the efficiency of pulsed light (PL) treatment applied in the control of microorganism growth in different fruits [7–11]. However, studies involving PL as radiation from the perspective of inducing resistance mechanisms are still scarce [12].

PL is a new, non-thermal technology, where a lamp containing an inert gas such as xenon emits high-frequency radiation pulses with wavelengths between 180 and 1100 nm [13]. This technology is used to sanitize the surfaces of fruit and vegetable by acting on microorganism cells and breaking and altering their DNA sequences, thus inhibiting the pathogen reproductive capacity [14]. Because of these positive effects of this technology on the control of pathogen growth in a wide variety of fruits, PL has been considered a new trend in the fruit industry to avoid or reduce the use of chemical fungicides. Moreover, the use of PL as a physical method can stimulate the production of phytochemicals in plant tissues to minimize the possible deleterious effects caused by radiation [15]. Thus, when fruit is subjected to abiotic stress, some metabolomics changes might be down- or upregulated in a variety of compounds related to fruit metabolism; this could even result in the production of new compounds linked to stress tolerance [16].

Metabolomics has been emphasized within the “omics” sciences, and efficiently evaluates a large part of the metabolites of an organism, both quantitatively and qualitatively, at a given time and in a specific situation [17]. Metabolomics can identify a change in the concentration of compounds involved in primary metabolism or the production of secondary metabolites. These metabolic alterations may arise from cellular lesions, metabolic adjustments to restore cellular homeostasis, or the synthesis and/or accumulation of metabolites in cellular pathways [18].

The objective of this study was to investigate the fungicidal or fungistatic effect of PL treatment on *F*. *pallidoroseum* on *Cucumis melo* var. Spanish, using chemometric tools to identify the possible metabolic changes in the defense mechanisms related to these effects (fungicidal or fungistatic). Where these biomarkers signify a normal or abnormal process or an adverse condition, a biomarker can be used to evaluate how well the organism responds to biotic and abiotic stress.

## Material and methods

### Chemical compounds

We used the following reagents: acetonitrile (PubChem CID: 6432), formic acid (PubChem CID: 284), methanol (PubChem CID: 887), sodium hypochlorite (PubChem CID: 23665760), MilliQ water (PubChem CID: 962), and liquid nitrogen (PubChem CID: 947).

### Plant material

Melons (*C*. *melo* var. Spanish) were obtained at the maturity stage (10–12°Brix; weight around 1.5 to 2.0 kg) from a commercial growing field of Norfruit Northeast Fruit, located in Mossoro-RN, Brazil (04°54’9.4”S, 37°21’59.9”W). The surfaces of mature melons were disinfected with 200 μg L^–1^ sodium hypochlorite solution for 2 min, rinsed, and allowed to dry. The melons were then artificially inoculated.

### Fruit inoculation

*Fusarium pallidoroseum* spore suspension was produced from pure colonies of the fungus at a concentration of 10^6^ spores mL^–1^. Melons were inoculated near the peduncles and adjacent regions with 100 μL inoculums of *F*. *pallidoroseum* (n = 5 inoculants). After inoculation, the melons were subjected to PL treatment.

### Disease control by PL treatment

After 12 h, there was already a pathogenic effect [19] on fruits inoculated with *F*. *pallidoroseum*. These fruits were irradiated in a PL chamber (XeMaticA-2LXL; SteriBeam® GmbH, Kehl, Germany) equipped with two xenon flash lamps and Teflon® transparent supports, which allowed the melons to be uniformly exposed over 360° by both lamps. There was a distance of 0.07 m between the fruit and lamps, and all internal sides of the chamber were covered by mirrors to improve the absorption of PL by the fruit. The lamps produced short-time pulses of 0.3 μs, where each pulse provided 0.3 KJ m^–2^ of energy, delivering broad-spectrum white light (200–1100 nm) with approximately 15%–20% of UV-continuous, according to the system’s built-in photodiode readings. The evaluation of disease control against *F*. *pallidoroseum* was carried out by dose screening, as follows: 0 (non-treated with PL), 6, 9, and 12 KJ m^–2^, according to the standard limits proposed [20]. After PL-screening, the inoculated and PL-treated melons were incubated in a box protected from light for 48 h at 28 ± 1°C with a relative humidity of 92% to ensure optimal conditions for mycelium growth [21]. The melons were then stored at 25 ± 1°C for 21 days. After this time, the severity of fungal disease was analyzed by measuring the radial lesion diameter in the inoculum region using a digital pachymeter (Digimess®, São Paulo, Brazil), and expressed as meters (m) and percentage of disease incidence (%). These analyses were performed using a randomized design, each treatment comprised eight replicates and data were subjected to analysis of variance (ANOVA) followed by Tukey’s test at 5% probability.

### Disease control promoted by a suitable PL dose against *F*. *pallidoroseum*

After PL-screening, an additional experiment was conducted using a suitable PL dose capable of controlling fungal disease in the fruit. To evaluate disease control, melons were inoculated with *F*. *pallidoroseum* under the same inoculation conditions described in the “Fruit inoculation” section. Inoculated melons were then irradiated with a PL dose of 9 KJ m^–2^ in the same instrumental and storage (48 h) conditions as those described in the “Disease control by PL treatment” section. The control group included inoculated and non-inoculated melons both without PL treatment. After 96 h of PL treatment, which is the time necessary for the fruit defense system to respond to stress conditions [7, 22–24], biological triplicates of each treatment were subjected to extraction processes for injection into a UPLC system.

### Extract preparation

Extracts were obtained according to the method of Moore et al. [25] with modifications. Pellets were extracted from different adjacent regions (0.005 m) to the inoculum at thicknesses approximately similar to that of the melon rind. Melon peel powder (1.00 g) was resuspended in 4 mL of MeOH/H_2_O (7:3 *v/v*). The homogenate was then subjected to sonication (UltraCleaner 1450, Unique®, Brazil) for 30 min, followed by centrifugation at 6,000 × *g* for 5 min. The pellet was re-extracted twice using 3 mL of MeOH/H_2_O (7:3 *v/v*), under the same ultrasound and centrifugation conditions. The supernatant was filtered through a 0.22 μm polytetrafluoroethylene (PTFE) membrane (Biotechla®, Bulgaria) and injected directly into the UPLC system.

### Chromatographic analysis by UPLC-QTOF-MS^E^

Analyses were performed on an Acquity UPLC (Waters, USA) system coupled to a Xevo QTOF mass spectrometer (Q-TOF, Waters). Separations were performed on a C18 column (Waters Acquity® UPLC C18; 150 mm × 2.1 mm, 1.7 μm). For metabolic fingerprinting, a 2 μL aliquot of the extract was subjected to UPLC analysis using an exploratory gradient with a mobile phase comprising deionized water (A) and acetonitrile (B), both containing formic acid (0.1% *v/v*). The extracts from melons were subjected to the exploratory gradient as follows: 2%– 95% for 15 min, at a flow rate of 500 μL min^–1^. Ionization was performed with an electrospray ionization (ESI) source in negative ion mode, in the range of 110–1200 Da. The optimized instrumental parameters were as follows: capillary voltage of –2800 V, cone voltage of –40 V, source temperature of 120°C, desolvation temperature of 330°C, flow cone gas of 20 L h^–1^, desolvation gas flow at 600 L h^–1^, and microchannel plate (MCP) detector voltage of –1900 V. The mode of acquisition was MS^E^, and the system was controlled using MassLynx 4.1 software (Waters Corporation). The extracts were injected in triplicate.

### Statistical analysis

The UPLC–MS data were processed using MassLynx® software (Waters Co., Milford, MA, USA), under the following conditions: retention time variation, ± 0.05 min; mass range, 110–1200 Da (accurate mass tolerance ± 0.05 Da); and noise elimination level, 5. For the structural identification of metabolites, molecular formulas were considered and *m/z* values were obtained from high-resolution spectra observed in the chromatogram at the higher intensity. The relative error is given in ppm for each formula. Margins of error of less than 10 ppm were considered for MS/MS study.

The structural proposals of molecules were performed using MS/MS data through the establishment of rational fragmentation patterns reported in the literature [26–29]. A list of peak identities was created using the retention time (t_R_) and error (*m/z*). For unidentified peaks, all possible molecular formulas were derived (elements C, H, N, and O, with a tolerance of 10 ppm, at least 2 C atoms) using the elemental composition tools available in MassLynx® software.

The UPLC–MS data analyzed by chemometrics were processed using MarkerLynx® software for Principal Components Analysis (PCA) and Orthogonal Partial Least Squared Discriminant Analysis (OPLS-DA) and S-Plots. S-Plots were obtained by OPLS-DA analysis to determine potential biomarkers that significantly contributed to the difference among groups [30–33].

## Results and discussion

### Growth of *F*. *pallidoroseum* under PL treatment

**Fig 1:**
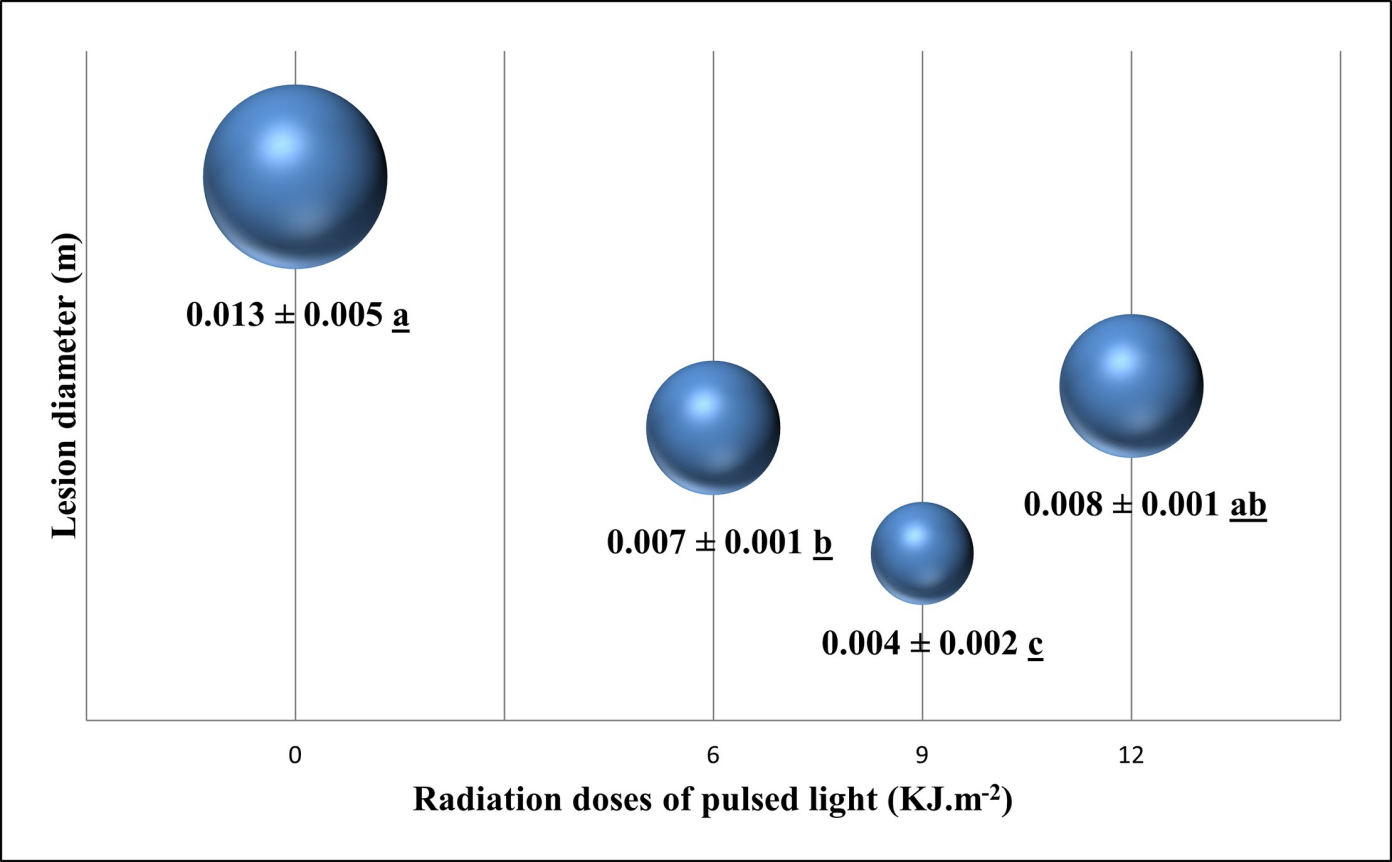
Screening of pulsed light (PL) doses to evaluate the lesion diameter (m) in melons inoculated with *Fusarium pallidoroseum*. Mean values followed by the same small letter did not differ significantly between PL treatments, by Tukey’s test at 5% probability.

The lesion diameter of fungal infection (Fig 1) and percentage of disease incidence were analyzed to determine the suitable PL treatment dose for the control or inhibition of *F*. *pallidoroseum* in melon fruit. The growth of the pathogen on inoculated melon fruit was strongly inhibited by PL treatment (Fig 1). This is corroborated, as shown in Fig 1, by a mean lesion diameter of 0.013 m, with a 100% incidence of fungal disease in melons without PL radiation.

Disease progression in the 6 KJ m^–2^ PL-treated group was significantly lower than that in the untreated group, with a significant reduction in lesion diameter (0.007 m) (Fig 1) and 62.5% incidence of fungal disease. Moreover, a dose of 9 KJ m^–2^ was the most effective of all the doses employed, and was associated with a mean lesion diameter of 0.004 m (Fig 1) and 33.3% disease incidence by *F*. *pallidoroseum*. This result indicates that the pathogen growth was inhibited or reduced, as shown in other studies that used PL technology [34, 35]; this behavior might encourage the fruit industry to avoid or significantly reduce the use of chemical fungicides.

The hormesis concept was used to explain the fungal behavior at the PL dose of 9 KJ m^–2^. Hormesis is a phenomenon in which low levels of potentially damaging radiation elicit beneficial responses, *i*.*e*., the physiological stimulation of beneficial responses in plants by low levels of stressors that otherwise elicit harmful responses. Hormetic doses of UV light (UV-continuous) radiation are involved in plant susceptibility toward diseases, and are capable of eliciting plant-resistance mechanisms such as the production of anti-fungal compounds [12, 36, 37]. UV-continuous radiation might also have a fungistatic effect promoted by phenolic compounds; they act as a barrier against both pathogenic attack and the diffusion of water and nutrients, which is important in pathogen growth [38].

A recent study compared the application of low-intensity UV-continuous and high-intensity PL sources as elicitors of hormesis in tomato fruit (*Solanum lycopersicum* ‘Mecano’) [12]. Curiously, these authors showed that postharvest hormetic treatment of tomato fruit with 16 pulses of PL (7.4 KJ m^–2^) with a spectral range (240–1050 nm) significantly delayed ripening along with inducing disease resistance to *Botrytis cinerea*, with a 41.7% reduction in disease progression compared to a 38.1% reduction in conventional low-intensity UV-continuous (254 nm) treatment at 0.37 KJ m^–2^. Thus, according to the authors, PL treatment, although rich in UV-continuous (broader spectral output), elicited the same pathways or responses as hormesis induced by conventional low-UV sources (narrower spectral range), making PL treatment more commercially attractive; it allows a substantial reduction in treatment time from seconds to microseconds [39, 40].

The last dose applied, 12 KJ m^–2^, corresponded to a disease incidence (37.5%) statistically equal to the treatment with 9 KJ m^–2^, but showed a mean lesion diameter twice as large (0.008 m) as that in the 9 KJ m^–2^ treatment (Fig 1). These results showed a possible damaging effect on melon, where an excess of PL can inhibit the fruit defenses against fungal disease, and that the dose of 12 KJ m^–2^ stipulated by the FDA [20] was limiting to the conditions assessed.

Based on these results, we can hypothesize that the PL treatment of 9 KJ m^–2^ applied here acted as a fungistatic agent inhibiting the mycelial growth of *F*. *pallidoroseum*; this behavior is linked to the probable hormetic effect associated with the induction of metabolite synthesis. Therefore, 9 KJ m^–2^ was chosen for identification of the metabolites produced.

### Putative metabolite identification by UPLC-QTOF-MS

**Fig 2:**
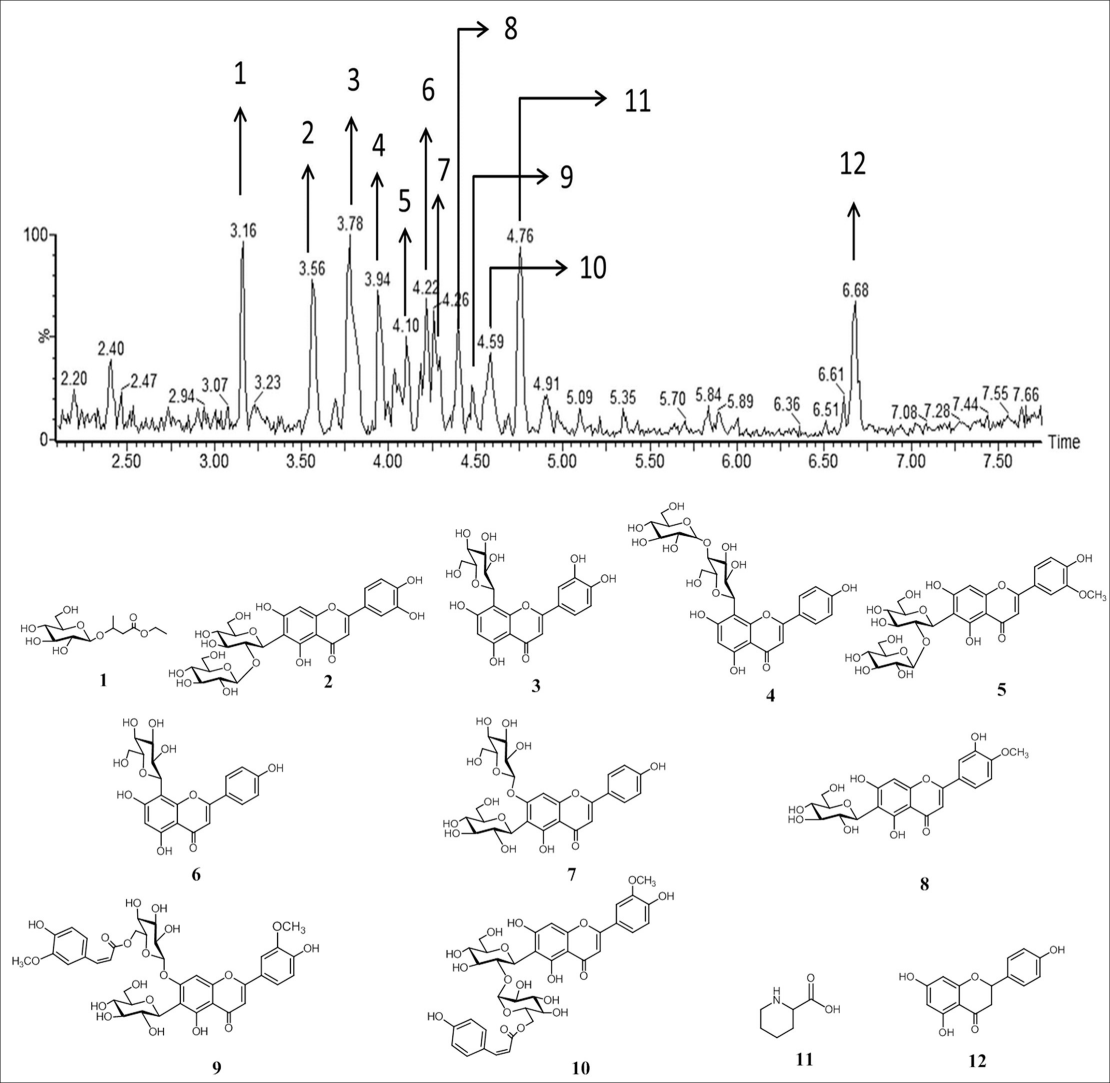
Base-peak chromatogram of melon inoculated with *Fusarium pallidoroseum* and subjected to pulsed light (PL) treatment at 9 KJ m^–2^.

The chemical profile of melon samples was established by analyzing the negative mode (ESI^–^) chromatograms (Fig 2) together with the mass spectra. The peaks were numbered according to their elution order, and the compounds were tentatively identified by interpretation of their MS and MS/MS spectra, acquired by QTOF-MS, along with data from the literature and open-access mass-spectra databases using MassLynx^®^. Table 1 lists the MS data of tentatively identified compounds, including the experimental and calculated *m/z* values for the molecular formula, error, and fragments obtained by MS/MS, as well as the proposed compound for each peak. In general, 12 metabolites of distinct chemical classes, organic acids, non-protein amino acids, and phenolics, were tentatively identified.

**Table 1:** Secondary metabolites tentatively identified by UPLC-QTOF-ESI-MS^E^ in melons treated with pulsed light (PL).

Peak	t_R_ (min)	Positive ion mode			Negative ion mode				Molecular formula	Compounds
		[M+H]^+^ Observed	[M+H]^+^ Calculated	Error (ppm)	[M-H]^-^ Observed	[M-H]^-^ Calculated	MS/MS	Error (ppm)		
1	3.16	295.1405	295.1393	4.1	293.1232	293.1236	131.0708	-1.4	C_12_H_22_O_8_	Hydroxybutanoic acid ethyl ester-hexoside
2	3.58	611.1619	611.1612	1.1	609.1454	609.1456	489.1016 429.0793 309.0857	-0.3	C_27_H_30_O_16_	Isoorientin-2′′- *O*-glucoside
3	3.78	449.1066	449.1084	-4.0	447.0936	447.0927	357.0532 327.0522 285.0197	2.0	C_21_H_20_O_11_	Orientin
4	3.96	595.1645	595.1663	-3.0	593.1486	593.1506	413.0811 293.0408	-3.4	C_27_H_30_O_15_	4′′ -*O*-glucosylvitexin
5	4.11	625.1752	625.1769	-2.7	623.1591	623.1612	443.1021 323.0592	3.4	C_28_H_32_O_16_	Isoscoparin 2″-*O*-glucoside
6	4.23	433.1118	433.1135	-3.9	431.0968	431.0978	341.0652 311.0542	-2.3	C_21_H_20_O_10_	Vitexin
7	4.28	595.1641	595.1663	-3.7	593.1498	593.1506	503.1073 473.9605 341.0679	-1.3	C_27_H_30_O_15_	Isovitexin-7′′- *O*-glucoside (Saponarin)
8	4.40	463.1236	463.1240	-0.9	461.1052	461.1084	371.0816 341.0660 299.0436	-2.8	C_22_H_22_O_11_	Diosmetin-6-*C*-glucoside
9	4.49	801.2205	801.2242	-4.6	799.2114	799.2086	461.1159 341.0681	3.5	C_38_H_40_O_19_	Isoscoparin 7-*O*-[6′′-feruloyl]-glucoside
10	4.59	771.2106	771.2136	-3.9	769.1970	769.1980	623.1699 443.0993 413.0831	-1.3	C_37_H_38_O_18_	Isoscoparin-2′′-*O*-(6′′′-ρ-coumaroyl)-glucoside
11	4.76	130.0866	130.0868	-1.5	128.0710	128.0712	84.0796	-1.6	C_6_H_11_NO_2_	Pipecolic acid*
12	6.62	273.0753	273.0763	-3.7	271.0605	271.0606	177.0180 151.0026 119.0489	-0.4	C_15_H_12_O_5_	Naringerin

* Compound tentatively identified in negative and positive ionization mode, with MS/MS in positive ion mode.

Peak **1** (t_R_ = 3.16 min) was tentatively identified as hydroxybutanoic acid ethyl ester-hexoside. This compound showed the ion *m/z* of 293.1232 [M-H]^−^in MS and the fragment ion *m/z* of 131.0708 [M-H-162]^−^in MS/MS, indicating a pattern loss of the hexoside moiety [41]. These classes of phenolic acids were also reported in melon by Mallek-Ayadi et al. [42] and other studies on *Cucumis sativus* showed the same presence of this class of compounds [43].

The mass spectrum of peak **2** (t_R_ = 3.58 min) showed the precursor ion *m/z* 609.1454 [M-H]^–^. The MS/MS spectrum showed fragment ions *m/z* 489.1016 [M-H-120]^–^, 429.0793 [M-H-180]^–^, and 309.0857 [M-H-180-120]^–^. The losses of 180 and 120 u are significant for diglucosides like sophoroside (1–2 linkages of two glucose molecules). Through the correlation of ions observed in MS and MS/MS, the compound was tentatively identified as luteolin-6-*C*-glucosyl-2′′-*O*-glucoside, also known as isoorientin-2′′-*O*-glucoside [44].

The mass spectrum of peak **3** (t_R_ = 3.78 min) presents a precursor ion *m/z* 447.0936 [M-H]^−^that exhibited fragment ions *m/z* 357.0532 [M-H-90]^–^, 327.0522 [M-H-120]^–^, and 285.0197 [M-H-162]^–^. The fragment ion *m/z* 285.0197 [M-H-162]^−^is the aglycone formed from the loss of the glycosidic group (Table 1 and Fig 2) [45]. Thus, the compound was tentatively identified as 8-*C*-glucosyl luteolin, also known as orientin [44]. Peaks **2** and **3** show moieties identified as luteolin are also found in *Cucumis sativus* L. as described by Abu-Reidah et al. [46].

Peak **4** (t_R_ = 3.96 min), showed in the mass spectrum as the precursor ion *m/z* 593.1486 [M-H]^–^, also exhibiting the fragment ions *m/z* 413.0811 [M-H-180]^−^and 293.0408 [(aglycone+41)-18)]^–^, which are characteristic of flavone *O*-glucosyl-*C*-glucoside, indicating the presence of sophoroside and apigenin as aglycone. This compound was characterized as apigenin-6-*C*-glucosyl-2′′-*O*-glucoside, also known as 4′′-*O*-glucosylvitexin or isovitexin-2′′-*O*-glucoside [44, 47].

Peak **5** (t_R_ = 4.11 min) showed in the mass spectrum as the precursor ion *m/z* 623.1591 [M-H]^−^and its respective fragment ions *m/z* 443.1021 [M-H-162+18]^–^, which suggested the loss of a glucose moiety, one unit of water, and the fragment ion *m/z* 323.0592 [M-H-120]^–^. These patterns of fragmentation indicate the presence of a diglucoside linkage; thus, compound **5** was tentatively identified as isoscoparin 2′′-*O*-glucoside [48].

Peak **6** (t_R_ = 4.23 min) in the mass spectrum presented the precursor ion *m/z* 431.0968 [M-H]^–^. In the MS/MS spectrum, it showed the fragments ions *m/z* 341.0652 [M-H-90]^−^and 311.0542 [M-H-120]^–^, indicating the presence of hexose as the monosaccharide and apigenin as aglycone. Therefore, the compound was tentatively identified as 8-*C*-glucosyl apigenin, also known as vitexin [44]. The peaks **4** and **6** both presents apigenin as aglycone; the presence of apigenin derivates are reported in *Cucumis* in the literature [46].

The mass spectrum of peak **7** (t_R_ = 4.28 min) showed the ion *m/z* 593.1498 [M-H]^−^and the fragment ions *m/z* 503.1073 [M-H-90]^−^and 473.9605 [M-H-120]^–^. This pattern of loss indicates the presence of a diglucoside similar to that in compound **5** and *m/z* 341.0679 [M-H-120-132]^–^; the loss of 132 Da represents a pentoside. Thus, the compound was tentatively identified as isovitexin-7′′-*O*-glucoside, also known as saponarin [49].

Peak **8** (t_R_ = 4.40 min) in the mass spectrum showed the precursor ion *m/z* 461.1052 [M-H]^−^and its fragments *m/z* 371.0816 [M-H-90]^–^, 341.0660 [M-H-120]^–^, and 299.0436 [M-H-162]^–^. The fragment ion *m/z* 299.0436 [M-H-162]^−^represents aglycone, formed by the loss of a glucoside moiety. Thus, the compound was characterized as diosmetin-6-*C*-glucoside [50]. Diosmetin derivate was also found in related studies [46].

The peak **9** (t_R_ = 4.49 min) with a mass spectrum showing the ion *m/z* 799.2114 [M-H]^−^and its fragments *m/z* 461.1159 [M-H-338]^–^, representing a loss of feruloyl plus a glucoside moiety, and *m/z* 341.0681 [M-H-feruloyl-glucoside-120]^–^. Thus, based on fragmentation, the metabolite was tentatively identified as isoscoparin 7-*O*-[6′′-feruloyl]-glucoside [48].

The peak **10** (t_R_ = 4.59 min) in the mass spectrum presents the ion *m/z* 769.1970 [M-H]^−^and its fragments *m/z* 623.1699 [M-H-146]^–^, showing a loss of the coumaroyl moiety; and *m/z* 443.0993 [M-H-coumaroyl-162-18]^–^, showing the successive losses of coumaroyl, glucoside, and a unit of water. The correlation of the observed ions indicates that the metabolite in question is isoscoparin-2′′-*O*-(6′′′-*p*-coumaroyl)-glucoside [4].

Negative and positive ionization modes were used to identify pipecolic acid, a non-protein amino acid (homolog of proline). Therefore, the peak **11 (**t_R_ = 4.76 min) showed the ions *m/z* 128.0710 [M-H]^−^and *m/z* 130.0866 [M+H]^+^ in the MS in the negative and positive ionization modes, respectively. Corroborating with chemical identification, the fragment ion *m/z* 84.0796 [M+H-COOH]^+^ was observed in the positive mode, referring to an aromatic core obtained from the cleavage of the carboxyl group (Table 1 and Fig 2) [51].

In peak **12** (t_R_ = 6.62 min) the ion *m/z* 271.0605 [M-H]^−^was observed with a fragmentation pattern in MS/MS showing the loss of ring B at *m/z* 177.0180 and by retro-Diels-Alder reaction at *m/z* 151.0026 and *m/z* 119.0489. Thus, it was tentatively identified as flavanone naringenin [52, 53]. This compound was also reported in other studies investigating phenolic compounds in *Cucumis melo* [42, 46].

### Chemometric analysis

PCA is a multivariate data analysis method that can synthesize data from an original matrix with many variables in a set of smaller orthogonal variables [54, 55]. We confirm that the chemometric analyses were centered in PL treatment with a dose of 9 KJ m^-2^, considered here as treatment for disease control in melon inoculated with *F*. *pallidoserum* (Fig 1).

**Fig 3:**
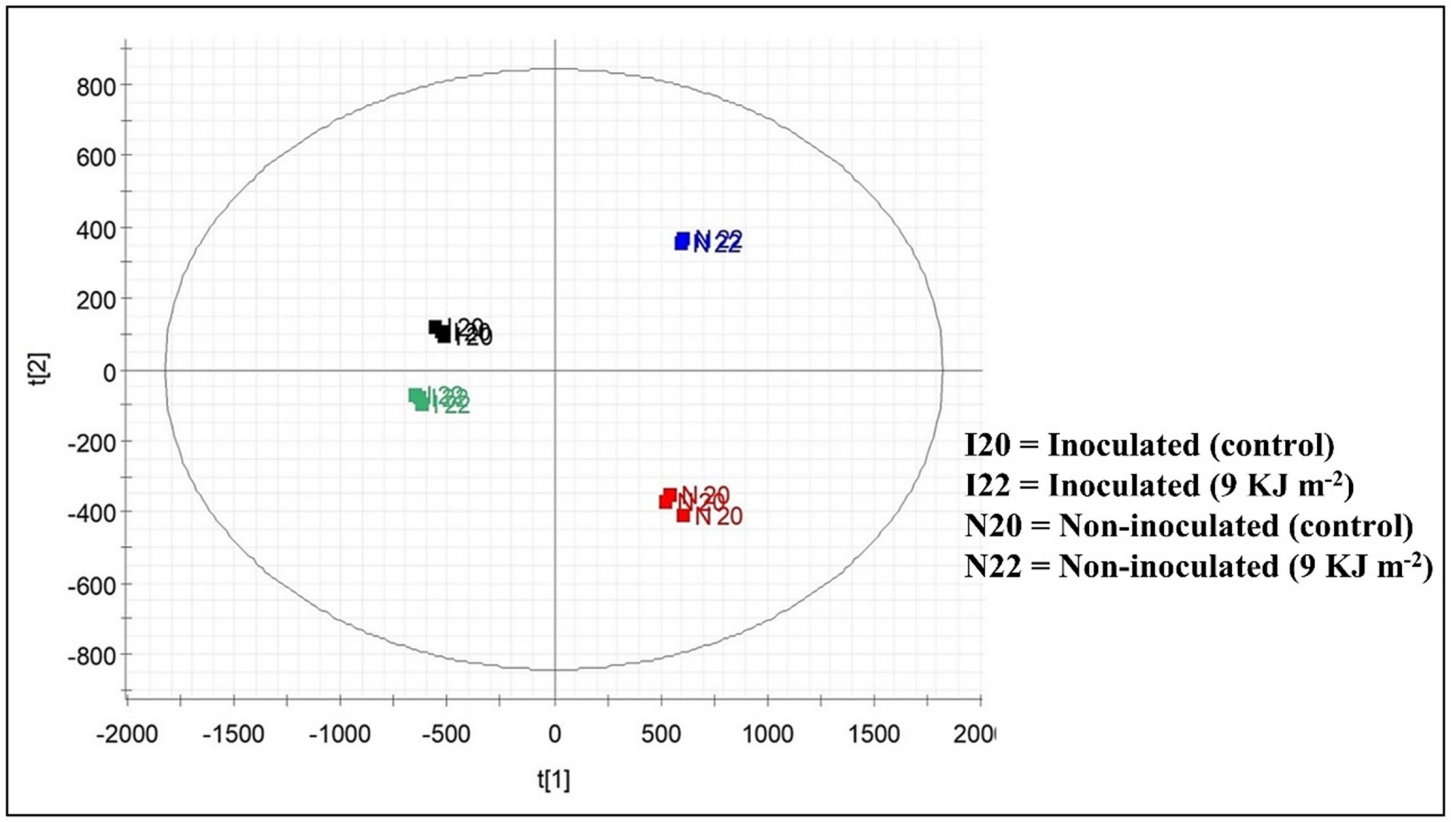
Discrimination of treatment groups by PCA-2D.

Therefore, PCA-2D was performed to discriminate between different treatment groups according to their metabolic profiles represented by the retention time and mass-to-charge ratio (rt-*m/z*) from the UPLC-QTOF-MS^E^ analysis (Fig 3). The PCA-2D showed perfect separation of all groups evaluated with 89% of the total cumulative variance in the diaxial axes PC1 and PC2 (R^2^X[1] = 0.7401 and R^2^X[2] = 0.1571), with a data noise level of 6%, indicating a robust model for data certainty. The formation of groups was related to the similarity between biological triplicates.

The PCA-2D in the PC1 showed the inoculated group (negative scores) and non-inoculated group (positive scores), whereas in the PC2 (positive scores), discrimination between the inoculated control and non-PL-treated group was observed. PC2 (negative scores) also showed the separation of inoculated PL-treated and non-inoculated groups (Fig 3). The separation of negative and positive groups in PC1 and PC2 was clearly linked to the differences between the metabolomic profiles [56]. Therefore, OPLS-DA chemometric analysis was applied to the UPLC-QTOF-MS^E^ data for comparing samples according to the metabolites that had influenced disease control against *F*. *pallidoroseum* in PL-treated melons. OPLS-DA is an analysis method used to study ions that contribute to experimental sample classification; the classification between the two groups in the OPLS-DA model can be visualized in the form of a score chart and scatter plot (S-plot).

**Fig 4:**
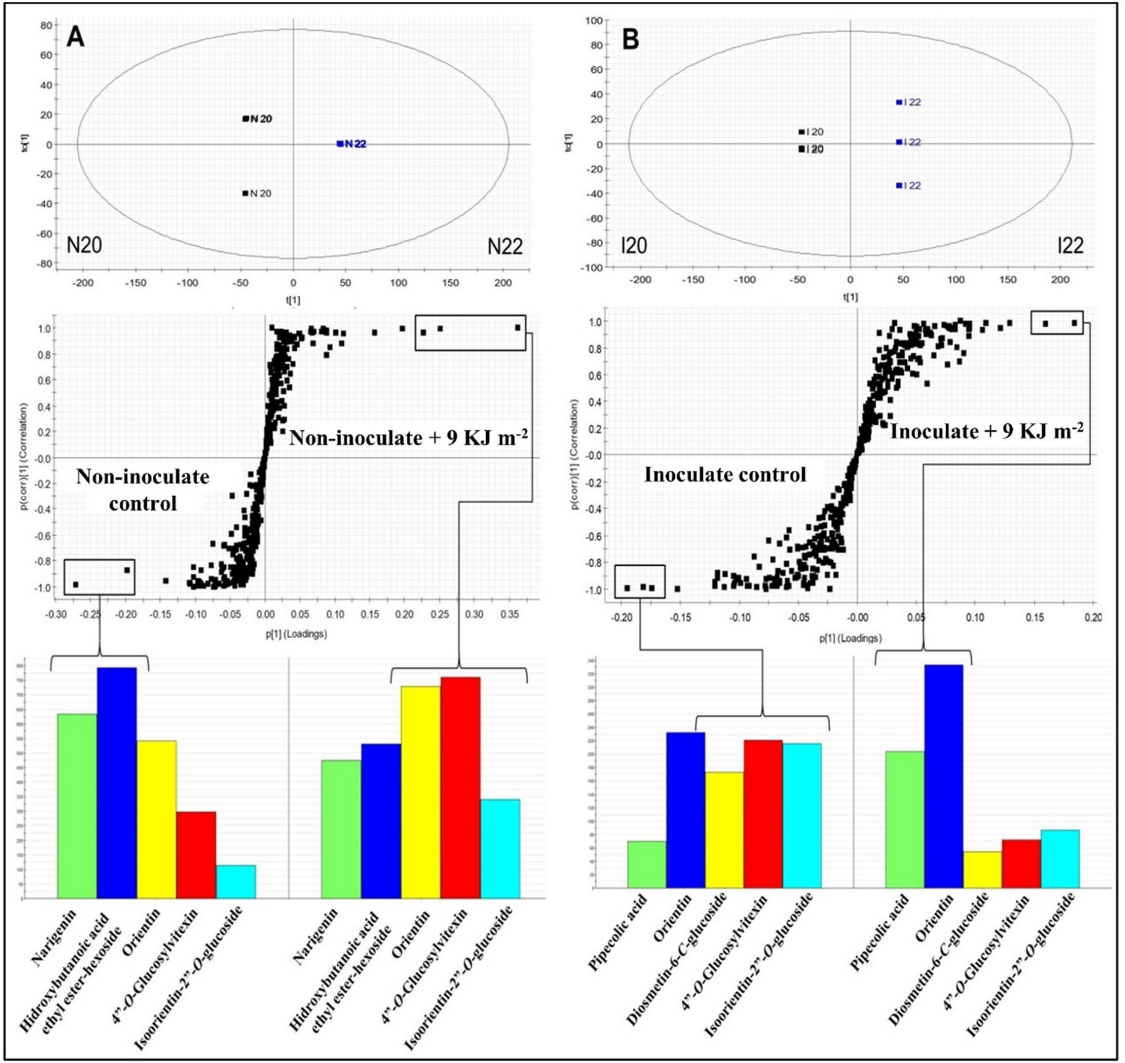
Orthogonal partial least squares discriminant analysis (OPLS-DA), S-Plot graphs and intensity of biomarkers between the non-inoculated control and the non-inoculated and pulsed light (PL)-treated melons (9 KJ m^–2^) (**A**). OPLS-DA, S-Plot graphs, and intensity of markers between the inoculated control and the inoculated and PL-treated melons (9 KJ m^–2^) (**B**).

Fig 4A summarizes the separation between the non-inoculated and non-treated fruit (control), and the non-inoculated fruit treated with PL (9 KJ m^–2^), with respect to the metabolic responses in the function of PL as abiotic stress, through OPLS-DA (R^2^X[cum] = 0.9232). The S-Plot generated from OPLS-DA, with variable influence on projection (VIP) > 1.0 and *p* < 0.05, showed the potential biomarkers between the treatments evaluated.

The control group showed the synthesis of principal secondary metabolites, such as naringenin (peak **12**), hydroxybutanoic acid ethyl ester-hexoside (**1**), isoorientin-2′′-*O*-glucoside (**2**), orientin (**3**), and 4′′-*O*-glucosylvitexin (**4**). However, the upregulation of naringenin (**12**) and hydroxybutanoic acid ethyl ester-hexoside (**1**) was highlighted (Fig 4A). Naringenin is a flavonoid that serves as a primer for more advanced flavonoid structures and as a substrate for glycosylation reactions. Hydroxybutanoic acid ethyl ester-hexoside has been previously described in other varieties of melon, such as Piel de Sapo, Galia, and Cantaloupe [41]. Interestingly, naringenin (**12**) and hydroxybutanoic acid ethyl ester-hexoside (**1**) were downregulated in melons treated with hormetic PL radiation, whereas specific flavonoid compounds such as orientin (**3**), 4′′-*O*-glucosylvitexin (**4**), and isoorientin-2′′-*O*-glucoside (**2**) were upregulated (Fig 4A). Thus, the upregulated compounds might be considered as biomarkers caused by PL treatment. The accumulation of flavonoid precursors, such as phenylalanine ammonia lyase, in vacuoles present in the epidermal and subepidermal mesophyll tissues of a fruit stimulate plant defense mechanisms under specific PL radiation conditions [36]. Many phenylpropanoids have been associated with induced disease resistance and disease control. According to Jung et al. [4] the flavonoids orientin, isoorientin-2′′-*O*-glucoside, and 4′′-*O*-glucosylvitexin are secondary metabolites involved in antioxidant activities against abiotic stress in rice leaves (*Oryza sativa* ‘Ilmi’) that were exposed to different conditions of LED-light radiation.

The differences between the inoculated control and inoculated PL-treated (9 KJ m^–2^) melons are shown in Fig 4B. Separation of the two treatment groups using the OPLS-DA graph (R^2^X[cum] = 0.8882) indicated the differences among groups according to their chemical profiles. The S-Plot obtained from the OPLS-DA graph, demonstrated the different potential biomarkers between groups for VIP > 1.0. Inoculation with *F*. *pallidoroseum* mainly induced the synthesis of glycosylated flavonoids, such as diosmetin-6-*C*-glucoside (**8**), isoorientin-2′′-*O*-glucoside (**2**), and 4′′-*O*-glucosylvitexin (**4**) (Fig 4B); this behavior is because of the presence of flavonoids at the infection site that are responsible for defense mechanisms against pathogens [57]. However, these metabolites, which are a natural response of fruit against the pathogen, were not sufficient to control the pathogen growth, as shown by the control treatment in Fig 1.

Biotic and abiotic stress might systemically regulate the defense mechanism by induced systemic resistance (ISR) or systemic acquired resistance (SAR) [58, 59]. PL treatment and inoculation with *F*. *pallidoroseum* led (by SAR) to the upregulation of two major biomarkers in melon, pipecolic acid (**11**) and orientin (**3**) (Fig 4B), which are present in high concentrations in the treatment. This indicated a change in the metabolic pathway, where the fruit preferentially used these specific compounds as biomarkers in response to the treatment [60]. The presence of pipecolic acid was verified in both of the groups that were inoculated with *F*. *pallidoroseum*. The presence of pipecolic acid in pathogen inoculation sites is associated with plant defense responses and acts as a regulator of inducible plant immunity [61]. Thus, when the PL treatment is applied, the pipecolic acid and orientin are upregulated, significantly achieving disease control (fungistatic effect), which is shown in Fig 1 with the 9 KJ m^–2^ treatment. Curiously, pipecolic acid is the supposed precursor of betaines, accumulated in the cytoplasm as a result of physiological plant responses to stress phenomena induced—in part—by adverse environmental conditions. These compounds, which are biochemically inert in the cell, are synthesized from some specific amino acids, such as serine, alanine, methionine, the non-protein amino acid such as γ-aminobutyric acid, and some cyclic amino acids, such as proline and pipecolic acid. The biosynthesis of betaines in the cytoplasm under abiotic stress conditions is mainly because of the action of methyltransferases, which utilize S-adenosylmethionine as a methyl group donor [60].

**Fig 5:**
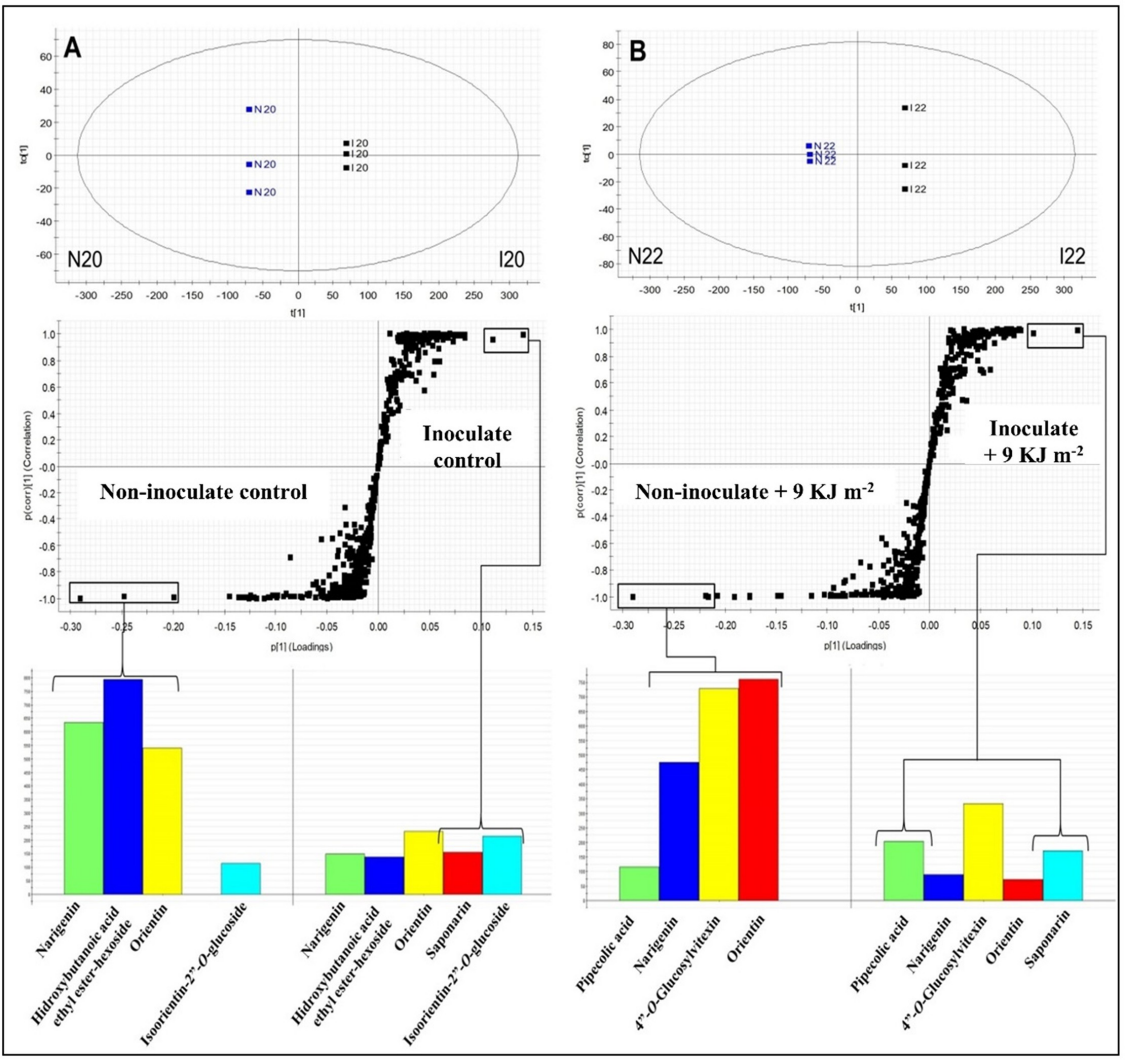
Orthogonal partial least squares discriminant analysis (OPLS-DA), S-Plot graphs, and intensity of biomarkers between non-inoculated and inoculated melons (**A**). OPLS-DA, S-Plot graphs, and intensity of markers between pulsed light (PL)-treated non-inoculated (9 KJ m^–2^) and PL-treated inoculated melons (9 KJ m^–2^) (**B**).

Fig 5A shows the results of the OPLS-DA graph (R^2^X[cum] = 0.9684) between the inoculated and non-inoculated groups in the function of *F*. *pallidoroseum* as a biotic stressor. The S-Plot originating from the OPLS-DA data demonstrated potential biomarkers in each group, with VIP > 1.0 and *p* < 0.05.

The number of compounds upregulated in non-inoculated melons was much higher than that in inoculated melons, and naringenin (**12**), hydroxybutanoic acid ethyl ester-hexoside (**1**) and orientin (**3**) were notably upregulated (Fig 5A). However, saponarin (**7**) was a biomarker present in the inoculated control melons and isoorientin-2′′-*O*-glucoside (**2**) showed slight upregulation in the same group (Fig 5A). Hydroxybutanoic acid ethyl ester-hexoside is a compound linked to amino acid groups, and some studies have indicated significant interconnections between different branches of amino acid metabolism and plant resistance to pathogens [62, 63]. Naringenin is a flavonoid that has shown metabolic activity associated with barley resistance to *F*. *graminearum* [64].

The results in Fig 5B show the separation of distinct groups, where the OPLS-DA plot (R^2^X[cum] = 0.9798) presents differences between the non-inoculated fruit and inoculated PL-treated fruits. The S-Plot obtained by the OPLS-DA graph indicates the potential markers for each group. The upregulation of flavonoids, such as naringenin (**12**), orientin (**3**) and 4”-*O*-glucosylvitexin (**4**), in non-inoculated PL-treated fruit (9 KJ m^–2^) was much higher than that in the inoculated PL-treated (9 KJ m^–2^) group (Fig 5B). The synthesis of flavonoids in detriment to the abiotic stress observed in PL treatment occurs owing to a change in the metabolic pathway of fruit in response to the stress received [8]. In contrast, inoculated and PL-treated melons accumulated a glycosylated flavonoid (identified as saponarin) (**7**) and pipecolic acid (**11**) as biomarkers [65]. Nevertheless, the presence of pipecolic acid in non-inoculated PL-treated (9 KJ m^–2^) melons (Fig 5B) shows that this metabolite might be synthetized by abiotic stress also. Saponarin is an antioxidant belonging to the flavones and is known to inhibit malonaldehyde formation in barley. In a normal reaction, malonaldehyde is formed from oxidized lipids on the surface of barley leaves by UV irradiation [66]. PL treatment of melons inoculated with fungi resulted in a response mediated by the synthesis of pipecolic acid in the cellular medium; this resulted in increased levels of betaines in the cell and induced the fruit immunity system [60].

## Conclusions

In this study, a PL dose of 9 KJ m^–2^ in melon inoculated with *F*. *pallidoroseum* controlled the disease promoted by this pathogen (fungistatic effect) and induced metabolic variation in the fruit defense system. Pipecolic acid (**11**) and orientin (**3**) were the two possible biomarkers associated with postharvest disease control against *F*. *pallidoroseum* in infected melons treated with PL radiation. This study also showed that fruit subjected separately to biotic and abiotic stresses demonstrated different metabolic responses, with a chemical profile in response to each stress. The compounds orientin (**3**), 4′′-*O*-glucosylvitexin (**4**), and isoorientin-2′′-*O*-glucoside (**2**) were only found in response to PL treatment. Our findings highlight that the application of PL technology provided control against the postharvest disease of *Cucumis melo* var. Spanish by directly controlling the growth of *F*. *pallidoroseum* through the synthesis/upregulation of specific compounds that acted as principal biomarkers of the defense system against pathogen. Thus, PL can be readily proposed as a new postharvest technological alternative to chemical fungicides; this could become an agriculture industry trend aimed at reducing the decay caused by *F*. *pallidoroseum* in *Cucumis melo* var. Spanish without leaving chemical residues during their export and storage.
